# Muslim perspectives on palliative care in perinatal and neonatal patients: a mini-review

**DOI:** 10.3389/fped.2023.1204941

**Published:** 2023-06-13

**Authors:** Abdullah B. Shoaib, Marissa Vawter-Lee, Charu Venkatesan, Ayman F. Soliman

**Affiliations:** ^1^ Department of Pediatrics, University of Cincinnati College of Medicine, Cincinnati, OH, United States; ^2^ Division of Neurology, Cincinnati Children’s Hospital Medical Center, Cincinnati, OH, United States; ^3^ Division of Pastoral Care, Cincinnati Children’s Hospital Medical Center, Cincinnati, OH, United States

**Keywords:** islam, muslim, palliative, end-of life, neonatal, perinatal, withdrawal, DNR

## Abstract

Muslims comprise nearly a quarter of the worldwide population, with significant populations in the United States, Canada, and Europe. As clinicians, it is important to be familiar with Islamic religious and cultural perspectives on medical treatment, life-prolonging measures and comfort and palliative care, but historically, this has been a gap in the literature. Recently, there have been multiple papers discussing Islamic bioethics, particularly in regards to end of life care in adults; however, there has been a lack of literature discussing the Islamic perspective on issues related to neonatal and perinatal end of life care. This paper uses clinical scenarios to review key relevant principles of Islamic law, discussing the primary and secondary sources used in formulating *fatawa,* including the Quran, *hadith*, *qiyas,* and ‘*urf*, and the importance of preservation of life and upholding of human dignity (*karamah*). Neonatal and perinatal scenarios are used to specifically explore the Islamic perspective on withholding and withdrawal of life-sustaining measures and determining what constitutes an acceptable quality of life. In some Islamic cultures the expertise of the patient's physician is given significant weight in making these judgments, and as such, families may appreciate frank assessment of the situation by the clinical team. Because of the various factors involved in issuing religious ruling, or *fatwa*, there is a wide spectrum of opinions on these rulings, and physicians should be aware of these differences, seek counsel and guidance from local Islamic leaders, and support families in their decision-making process.

## Introduction

Muslims comprise nearly a quarter of the world's population (1), with sizeable populations in the United States, Canada, and Europe that continue to grow (2). As clinicians, it is important to be familiar with religious and cultural perspectives on potentially sensitive topics. Historically, literature on Islamic medical ethics has been sparse (3). Within the past decade, there has been increasing interest in Islamic bioethics and palliative care, predominantly in adults (4–6). However, there remains a paucity of literature discussing the Islamic perspective on issues related to neonatal and perinatal palliative care.

Having a basic understanding of the principles of Islamic law is vital in fully appreciating the nuances of the Islamic perspectives on neonatal and perinatal palliative care issues. Islamic law, or *shariah*, is founded on two primary sources—the Quran, believed by Muslims to be the incorruptible word of God, and the *sunnah,* actions and sayings of Prophet Muhammad, whom Muslims believe to be the final messenger of God, collected in books of *hadith* (3). Two secondary sources are also used- *ijma'a*, which is consensus amongst Muslim religious scholars, and *qiyas*, which refers to reasoning based on the textual evidence in the primary sources (3). Muslim religious scholars who have completed extensive studies in interpreting these texts can be granted the title of *mufti*, and may issue non-binding scholarly opinion, or *fatwa* (pl. *fatawa*) on a religious matter or on a specific situation (3, 7). The act of researching the circumstances and issuing a *fatwa* is referred to as *ifta*. In contemporary matters with no clear textual basis for a ruling, the *mufti* must use *qiyas* (reasoning by analogy), ‘*aql* (intellect), and also consider ‘*urf* (the culture of the people), in deriving a *fatwa* (3).

In Muslim-majority countries, committees of religious scholars may meet regularly to discuss certain topics and issue a joint *fatwa* that can be used as a basis for legislation (5, 8). In non-Muslim majority countries, different individual scholars or Muslim organizations may issue their own *fatawa* in books, on television, or even online (3). With the absence of a central religious clergy and the multitude of factors involved in issuing *fatawa*, there often exists a wide spectrum of opinions on any given manner, and Muslims may decide to follow the *fatwa* that is issued by a scholar or organization that has the most similar background to them, the scholar whom they trust the most, or the *fatwa* that puts their mind at ease (3, 9).

We present three cases that explore Islamic perspectives on different aspects of neonatal and perinatal palliative care.

## Case 1: minimum age of viability—a case study in using Islamic sources to address contemporary issues

A neonate, born at 23 weeks gestation to a Muslim family, is apneic and asystolic at birth. Prior to delivery, parents had discussions with the Palliative Care team regarding the spectrum of treatment options available, and requested life-sustaining measures to resuscitate their child. What does Islam say about resuscitation of periviable neonates?

Contemporary discussions in the Muslim world on the resuscitation of periviable neonates is an excellent case study to better appreciate the process by which Islamic scholars issue a *fatwa*. Al-Alaiyan's paper calls for a national lower limit of viability and discusses the poor short-term and long-term outcomes of patients who are born significantly premature, both in Saudi Arabia as well as globally (8). In 2008, the General Presidency of Scholarly Research and *Ifta* in Saudi Arabia issued a *fatwa* stating: “In the case of infants born at less than 6 lunar months, two specialist physicians could study the infant’s clinical condition and based on their opinion, the infant could be offered full resuscitation if it is beneficial to the infant, or he or she can be left without intervention to die but should not be deprived of nutrition or fluid” (10). The determination of 6 lunar months (or roughly 25 weeks) does not come explicitly from primary Islamic texts, but comes from religious scholars' interpretation of verses from the Quran (8, 10, 11).

This *fatwa* was shaped in part by primary sources, the intellect (‘*aql*) of experts detailing poor outcomes for preterm neonates, as well as the culture and customs of the local population (‘*urf*) (8, 10). The subject of using and interpreting primary Islamic sources is outside the scope of this paper, but suffice to say that the lack of explicit primary textual support for this opinion is a point of contention surrounding this particular *fatwa*.

Some have raised concerns that discussions surrounding formulation of this *fatwa* had limited input from medical specialists. This is a common criticism regarding *fatawa*, particularly related to those regarding contemporary issues in healthcare, despite the inclusion of medical experts on many *fatwa*-issuing bodies (12–15). In addition, the ambiguity in what constitutes benefit, and how this is determined may be challenging for families and physicians to navigate (10, 16). However, others may appreciate this ambiguity, as it permits physicians the latitude to make decisions on a case-by-case basis (16).

This *fatwa* also appears to be shaped by customs and circumstances (‘*urf*) in Saudi Arabia, which raises concerns that application of this *fatwa* for Muslims living outside of Saudi Arabia would not be appropriate. Al-Alaiyan's paper cites the significant gaps in neonatal care in Saudi Arabia and how their delivery of neonatal care could influence how aggressively periviable neonates are resuscitated (8). The fact that this *fatwa*, which is non-binding and issued on a case-by-case basis, implies that parents have minimal or no input in decisions to resuscitate a neonate, may reflect cultural differences in the physician-patient relationship in Saudi Arabia (16, 17). The notion of limited or no parental input in decisions regarding withholding resuscitation, would immediately raise concerns to most physicians throughout the world, regardless of religious beliefs. The absence of any explicit directions from primary Islamic sources, the lack of scholarly opinions on this matter from outside of Saudi Arabia, and the lack of scholarly consensus (*ijma’a)* are all other significant shortcomings of this view (3). This represents an area in which further research should be done by Islamic bioethicists.

## Case 2: neonatal quality of life, *idrak*, and feeding tubes

An infant is born at 37 weeks to a Muslim family, and admitted to the neonatal intensive care unit (NICU) with bacterial meningitis and sepsis. As a sequela from the meningitis, the infant suffers severe cerebral encephalomalacia and intractable seizures. The family decides to compassionately extubate the patient, but the patient continues to breathe spontaneously on nasal cannula. The patient is on total parenteral nutrition (TPN), and family is considering placement of a gastrostomy tube, and asks about the patient's “quality of life” with placement of a feeding tube.

The patient's perceived quality of life is an important factor for physicians and families when deciding to redirect goals of care. A *fatwa* issued by the Association of Muslim Jurists of America (AMJA) states that withdrawal of life-sustaining therapies is permissible “if resumption of a stable, dignified life is not expected” (19). Though this is a vague, it does accurately reflect the fact that acceptable quality of life varies widely from family to family. However, there has been little guidance from Islamic scholars on a formalized quality of life assessment for neonates, and some families may look to spiritual sources for guidance on this issue.

Padela and Mohiuddin make the argument that, from the Islamic paradigm, the purpose of life is meaningful obedience and worship of God. As such, the authors state that the decision to medically intervene should reflect whether or not the patient would be able to return to a state in which they have the ability to meaningfully perform such acts, or in the case of the neonate, return to a state such that they are able to develop this ability (7). This stance has been criticized by Rady and Verheijde, who state that this metric is meant as a threshold to determine accountability for one's actions and not as a metric to determine the value of a life (20). In addition, Padela and Mohiuddin give the caveat that this metric should only apply to patients who, prior to their illness, were expected to develop the ability to perform meaningful acts of worship. However, in the NICU, the underlying etiology for patient's symptoms are not always well-elucidated, making this framework very difficult to apply.

The European Council of Fatwa and Research (ECFR) uses another approach to determine an acceptable quality of life. This approach relies on the concept of *idrak*, which refers to the ability of someone to perceive and respond to their surroundings. A *fatwa* issued by ECFR allows for withdrawal of life-sustaining treatment in situations where this ability is permanently lost (16). This is especially challenging to predict in the neonatal period because of the remarkable potential of the neonatal brain to adapt to neurological injuries through neuroplasticity. As such, it is difficult to predict neurological outcomes exclusively based on imaging or the results of physical examination, particularly in rare conditions.

This case also brings up the issue of feeding/hydration at the end of life. The *fatawa* cited earlier in discussions of the permissibility of withdrawal of life-sustaining measures predominantly agree that even if further life-sustaining measures are not pursued or life-sustaining treatment is being withdrawn, patients should continue to receive hydration and nutrition as long as clinically able, as withholding nutrition could potentially lead to death by starvation, which is a sin (5). However, Sultan offers the caveat that the initiation of artificial nutrition and hydration, such as through the placement of a gastrostomy tube or total parenteral nutrition (TPN), can be interpreted as a medical treatment, as it is manufactured and designed as something “artificial”, and as such, may not rise to the level of being religiously mandatory, depending on the clinical scenario (4). Thus, artificial nutrition is viewed by some as an essential food that cannot be stopped, and viewed by others as a lab manufactured medication that can be stopped. In our patient's case, if after discussions with the medical team the family decides that the patient's quality of life, including being dependent on a gastrostomy tube, is not acceptable, it would be permissible to defer gastrostomy tube placement. This topic is difficult to navigate for physicians and families, and it may be best to confer with *imams* and chaplains to help families come to the decision they feel is most in line with their values.

## Case 3: withholding and withdrawing life-sustaining treatment while preserving life, protecting *karamah*, and minimizing suffering

A neonate, born at 38 weeks to a Muslim family, is admitted to the NICU for seizures. Genetic testing confirms that the infant has neurodegenerative condition. After confirming the diagnosis, family decides to make the patient DNR/DNI. After an acute decompensation, family decides to withdraw life-sustaining care. What is the Islamic perspective on DNR/DNI orders, and withdrawal of life-sustaining care?

This case illustrates one of the most challenging discussions that physicians and parents face in the NICU—redirecting goals of care and not escalating medical interventions. Although the majority of the discussions in the Islamic palliative care literature center around adult patients, the underlying principles are the same for neonates (4, 5, 21, 22). Broadly speaking, Muslim religious scholars state that seeking medical treatment is mandatory if forsaking curative treatment will lead to loss of life or limb, and if the medical intervention is nearly guaranteed to prevent this harm from occurring (21, 23). This follows from one of the cardinal goals of *shariah*, which is to preserve life and to minimize harm (3).

However, some of the interventions that could be offered to this patient, such as ketogenic diet, will not reverse injury and are not guaranteed to be effective, nor do they act to directly prevent proximal causes of death. In these gray areas, there exists a spectrum of opinions. Generally, the status of seeking treatment (mandatory, recommended, optional, disliked, forbidden) is intimately linked to the likelihood that the intervention will be efficacious and the risk of the intervention (21, 23). For instance, seeking out medical treatment may be recommended in situations where the benefit is likely with little to no adverse effects, such as using antibiotics to treat pneumonia (5, 21). On the other hand, pursuing medical treatment may be discouraged if the negative effects of the treatment outweigh possible benefits (4, 5, 21).

In addition to protecting life, one of the goals of Islamic law is to also protect *karamah*, or dignity, of a patient (16). As in this patient case, families may feel that signing a “do not resuscitate” or “do not intubate” order is the appropriate decision if escalating medical therapy is deemed unlikely to improve the patient's overall clinical status, and will cause perceived loss of the patient's dignity (16, 24). This view is supported by multiple *fatawa,* including one from the Permanent Committee for Research and Fatwa in Saudi Arabia, which in 1989, issued a *fatwa* which allows for non-resuscitative matters if the patient or their caretaker has made a directive to not perform resuscitative measures, and three physicians agree that the patient is unsuitable for resuscitation (5, 25). This same *fatwa* also states that if three physicians feel that resuscitative measures are inappropriate, further interventions do not need to be taken, and the will of the patient and their family do not need to be taken into account (5). Like the *fatwa* discussed in the first case, this may reflect the culture of healthcare in Saudi Arabia.

Islamic religious scholars apply the same principles to withdrawing life-sustaining interventions as withholding life-sustaining treatments (4, 16, 26). As is the case with withholding life-sustaining treatments, the two factors that clinicians and family must balance are the preservation of life and dignity with minimizing harm*.* Earlier scholarly opinions, such as a *fatwa* from the Permanent Committee for Research and Fatwa in Saudi Arabia in 1983, have permitted withdrawal of life-sustaining measures in patients who have severe irreversible neurological injury (21). However, more recent *fatawa* by eminent religious scholars and organizations from the United States, India, Saudi Arabia, and other countries also permit withdrawal of life-support and medical treatment if the patient is terminally ill,, there is no hope for recovery, or if continued treatment will bring harm (5, 16, 19, 21, 22, 25, 26). Though this view represents the majority opinion from multiple different cultures, there still exist conflicting opinions about the permissibility of withdrawing life-sustaining measures (16).

## Discussion

Islamic scholars and physicians continue to tackle contemporary bioethical issues, including those surrounding palliative care in neonates. There have been attempts to suggest a minimum age of viability from Islamic texts, with a *fatwa* from Saudi Arabia suggesting 25 weeks of gestation (8, 10). However, the rapid changes in the field of neonatology and associated outcomes, the lack of explicit primary texts, and differences in the healthcare system in the United States and Saudi Arabia make this a very complex topic, with a wide spectrum of opinions, even amongst Muslim physicians and scholars (11, 18, 27). On the other hand, there is a strong opinion amongst Islamic scholars from many countries with large Muslim populations that withdrawing or withholding life-sustaining therapy is permissible in certain situations where treatment may be unlikely to bring benefit, or if there is expected to be poor long-term outcomes or quality of life, as this can be seen as protecting the patient's *karamah* or dignity (16, 24). Determination of what is a minimum acceptable quality of life can vary among families. However, one opinion that has been put forth by Islamic scholars is that a minimum quality of life constitutes having *idrak*, or the ability to sense their surroundings, and meaningfully react to these surroundings (16).

There exists a significant spectrum of Islamic opinions on these topics, all of which have some grounding in Islamic sources and principles. Families' own backgrounds and experiences may shape which *fatwa* resonates most with them. It is vital to understand that these differences exist, why they exist, and to support families no matter which decisions they decide to make.

In the experience of the Muslim chaplain at our institution (AFS), Muslim families lean on religion during times of difficulty, even if they are not actively practicing. In addition, decision-making usually involves discussions with multiple people, often including family and sometimes *imams* from their country of origin. This poses a challenge because some *imams* are not well-versed in matters regarding end-of-life care, and thus, may view withdrawal of life-sustaining treatment as sinful. Many families may not know about the different opinions on withdrawal of life-sustaining treatment, and informing them about these different opinions may empower families to make decisions about which they feel conflicted, such as pursuing withdrawal of life-sustaining treatment. Though not always possible, Muslim families appreciate having these goals of care discussions with Muslim healthcare workers, due to a perceived common value system, and may be more open to expressing their sincere thoughts with them.

## Conclusion

Further collaboration between medical experts, Islamic scholars and leaders, and bioethicists, can provide more well-informed *fatawa*, taking into account both the religious aspect of these difficult decisions, along with the medical challenges that arise in these cases. Having *fatawa* from outside the Arab world, where cultural practices and medical infrastructure may be different, would also help provide insight to physicians practicing in different parts of the world. This paper investigated this topic from the Sunni perspective, but further literature into the Shi'ite perspective, who comprise nearly 15%–20% of the worldwide Muslim population, is also needed. Finally, medical providers should reach out to *imams* and chaplains to ensure Muslim families have support from people who are well-informed about the Islamic perspectives on relevant issues. Respecting differences, and understanding that these differences may be informed by different interpretations of religious rulings, is of utmost importance in supporting Muslim patients and their families.
